# XPD localizes in mitochondria and protects the mitochondrial genome from oxidative DNA damage

**DOI:** 10.1093/nar/gkv472

**Published:** 2015-05-12

**Authors:** Jing Liu, Hongbo Fang, Zhenfen Chi, Zan Wu, Di Wei, Dongliang Mo, Kaifeng Niu, Adayabalam S. Balajee, Tom K. Hei, Linghu Nie, Yongliang Zhao

**Affiliations:** 1 Key Laboratory of Genomics and Precision Medicine, Beijing Institute of Genomics, Chinese Academy of Sciences, Beijing 100101, China; 2 University of Chinese Academy of Sciences, Beijing 100049, China; 3 Hebei North University, Zhangjiakou 075000, China; 4 REAC/TS, Oak Ridge Associated Universities, Oak Ridge Institute of Science and Engineering, Oak Ridge, TN 37830, USA; 5 Center for Radiological Research, Department of Radiation Oncology, Columbia University Medical Center, New York, NY 10032, USA

## Abstract

Xeroderma pigmentosum group D (XPD/ERCC2) encodes an ATP-dependent helicase that plays essential roles in both transcription and nucleotide excision repair of nuclear DNA, however, whether or not XPD exerts similar functions in mitochondria remains elusive. In this study, we provide the first evidence that XPD is localized in the inner membrane of mitochondria, and cells under oxidative stress showed an enhanced recruitment of XPD into mitochondrial compartment. Furthermore, mitochondrial reactive oxygen species production and levels of oxidative stress-induced mitochondrial DNA (mtDNA) common deletion were significantly elevated, whereas capacity for oxidative damage repair of mtDNA was markedly reduced in both XPD-suppressed human osteosarcoma (U2OS) cells and XPD-deficient human fibroblasts. Immunoprecipitation-mass spectrometry analysis was used to identify interacting factor(s) with XPD and TUFM, a mitochondrial Tu translation elongation factor was detected to be physically interacted with XPD. Similar to the findings in XPD-deficient cells, mitochondrial common deletion and oxidative damage repair capacity in U2OS cells were found to be significantly altered after TUFM knock-down. Our findings clearly demonstrate that XPD plays crucial role(s) in protecting mitochondrial genome stability by facilitating an efficient repair of oxidative DNA damage in mitochondria.

## INTRODUCTION

Xeroderma pigmentosum (XP) is a human autosomal recessive disease and is characterized by seven complementation groups from A through G. XPD/ERCC2 (Rad3 in *Saccharomyces* *cerevisiae*), an ATP-dependent helicase, belongs to RAD3/XPD helicase subfamily (1). XPD protein serves as a part of TFIIH basal transcription factor complex and plays essential roles in both transcription and nucleotide excision repair (NER) (2,3). During the NER process, XPD is essential for DNA unwinding at the sites of bulky adducts through its helicase activity (4).

Mutations in *XPD* can cause several human diseases: Xeroderma pigmentosum group D (XP-D), XPD patients combined with Cockayne syndrome (XP/CS), Trichothiodystrophy photosensitive (TTDP) and Cerebro-oculo-facio-skeletal type 2 (COFS2). XP is the first-identified human disorder associated with DNA repair deficiency with a 2000-fold higher risk for skin cancer incidence upon sunlight exposure than normal population. Neurological abnormalities have also been reported in severely affected human XP patients. UV hypersensitivity and neurodegeneration are the common features of XPD-related human disorders (5–7). The data from *in vivo* mouse models consistently demonstrated that XPD-deleted mice are embryonically lethal, whereas XPD-mutated TTDP mice exhibit many pathological features as TTDP patients including hair brittle, smaller size, premature ageing and reduced lifespan (8,9). Additionally, both human and mouse TTDP cells are hypersensitive to acute oxidative stress, illustrating the critical role of XPD protein in repairing the DNA damage sites induced by oxidative stress (10).

Reactive oxygen species (ROS) can generate multiple DNA lesions, including oxidized DNA bases, abasic sites, and single- and double-strand breaks. Mitochondria are considered to be the ‘power house’ of the cell and the process of ATP generation by oxidative phosphorylation involves the transport of protons across inner mitochondrial membrane by electron transport chain. Due to an increased level of ROS generation in mitochondria, mtDNA is constantly exposed to a high level of oxidative stress leading to mtDNA damage accumulation and mutations that are thought to play an important role in the aging process (11). Therefore, functional integrity of mitochondrial genome relies on the existence of efficient base excision repair (BER) pathway that specifically removes oxidative stress-induced mtDNA damage. NER, a highly conserved pathway, plays a critical role in the maintenance of genomic stability by repairing bulky DNA adducts and all of the seven XP genes (XPA through XPG) play crucial roles in damage recognition, incision and excision steps of NER. Additionally, two other genes of CSA and CSB responsible for Cockayne syndrome group A and B patients are the integral components of a special sub-pathway of NER known as transcription coupled repair that preferentially removes DNA lesions from the transcribing strand of active genes. Although UV-induced NER is demonstrated to be largely defective in mitochondria, crucial NER factors such as CSA, CSB and Rad23a have been found localized in mitochondria (12). Additionally, one of the human RecQ helicases, RecQL4, which plays a role in NER through its association with XPA, has also been detected in mitochondria (13,14). A recent report of mitochondrial dysfunction in XPA cells clearly indicates that NER genes are also crucial for mitochondrial genome stability (15).

Induction of endogenous oxidative lesions has been demonstrated to be much higher in mtDNA than nuclear DNA due to a high level of ROS generation in mitochondria (16,17). Among the XPA, XPB and XPD cell lines tested, XPD-deficient lymphoblastoid cells showed the highest sensitivity to hydrogen peroxide induced oxidative DNA damage (18). Consistent with this, a recent finding suggested that XPD, one of the subunits of the basal transcription factor TFIIH, is strictly dedicated to DNA repair activity (19). Observation of enhanced sensitivity of XPD cells to oxidative DNA damage raises an interesting possibility that XPD may play a crucial role in the repair of oxidative DNA damage not only in nuclear DNA but also in mtDNA. Since some of the NER factors such as CSA and CSB are localized in mitochondria and participate in mitochondrial BER, we wished to investigate whether or not XPD also localizes in mitochondria and mediates the repair of oxidative damage in mtDNA.

In this study, we provide the novel evidence that XPD protein is localized in mitochondria and XPD deficiency leads to an increased level of mitochondrial ROS and mtDNA deletions suggestive of mitochondrial dysfunction. Strikingly, XPD deficiency also greatly reduces the capacity to repair hydrogen peroxide induced oxidative damage in mtDNA. Further, XPD protein in mitochondria physically interacts with TUFM, a mitochondrial Tu translation elongation factor. Mitochondrial dysfunction similar to that found in XPD-deficient cells was also observed in TUFM knock-down cells illustrating the importance of both XPD and TUFM proteins for mitochondrial DNA stability. Our findings suggest that XPD through its interaction with mitochondrial TUFM plays a crucial role in the maintenance of mitochondrial stability by protecting the mtDNA from endogenous and exogenous oxidative DNA damage accumulation.

## MATERIALS AND METHODS

### Cell culture and antibodies

The human osteosarcoma cell line U2OS and human embryonic kidney cell line HEK293 were grown in Dulbecco's modified Eagle's medium (DMEM, GIBCO) supplemented with 10% fetal bovine serum (FBS, Hyclone) at 37°C in humidified 5% CO_2_ incubator. Human dermal fibroblasts derived from normal (NHDF, Clonetics) and XPD patient cells (GM00434) were grown in Dulbecco's Modified Eagle Medium/Ham's F-12 (DMEM/F-12; GIBCO) supplemented with 15% FBS, non-essential amino acids, essential amino acids, L-Glutamine, Vitamins and sodium pyruvate (GIBCO).

The following primary antibodies used for western blotting analysis were as follows: mouse anti-Flag (sigma), rabbit anti-XPD (Cell Signaling), goat anti-Lamin B for detecting nuclear protein (Santa Cruz); mouse anti-GAPDH for detecting cytoplasmic protein (Millipore); rabbit anti-VDAC for mitochondrial outer-membrane protein, mouse anti-SMAC for mitochondrial intermembrane space protein, rabbit anti-TUFM (Sigma) and mouse anti-β-actin (Cell Signaling).

### Establishment of XPD overexpressed or silenced cell lines

U2OS and HEK293 cells were transfected with pcDNA3.0/Flag-XPD and pcDNA3.1/Flag-HA-XPD plasmid DNA and selected with 400 μg/ml G418 or 800 μg/ml Hygromycin B, respectively. XPD-overexpressed U2OS or HEK293 cells were then established by screening the antibiotics-resistant clones by western blotting. XPD knock-down U2OS cells were acquired using lentiviral vector-mediated shRNA approach. In brief, *XPD* shRNA (shXPD) sequence: GGCATCTACAACCTGGATGACCTGAAGGC (Origene) and shControl sequence GAAGAGGACAC GCCTTAGACT were cloned into pU2-FH lentiviral vector (kindly provided by Dr Yunfeng Feng). 293Tn packaging cells were co-transfected with shXPD or shControl lentiviral construct and packaging plasmid mix. The viral supernatant was collected at 48 h post transfection. The U2OS cells were then infected by the viral particles and selected with 2 μg/ml Puromycin. The siRNA for *TUFM*: GCGGCUCAUGUGGAGUAUATT and siRNA control: UUCUCCGAACGUGUCACGUTT were designed and purchased from GenePharma (Shanghai, China). U2OS cells were transfected with siRNA using Rfect siRNA transfection reagent (Bio-trans, China) following the recommended procedures.

### Immunofluorescence staining

Cells were seeded onto chamber slides for 24 h, rinsed with phosphate buffered saline (PBS) and incubated with 0.5 μM of MitoTracker Red CM-H2XRos (Molecular Probes) for 30 min. Cells were washed with PBS and fixed in 4% paraformaldehyde. Cells were permeabilized with 0.1% Triton X-100 for 10 min, and then cells were blocked with 5% non-fat milk (Santa Cruz). Cells were incubated with anti-Flag (Sigma) antibody at 4°C overnight. After washing three times with PBS, cells were incubated with fluorescence-conjugated Texas Green Goat anti-Mouse IgG antibodies (Vector laboratories) at room temperature for 2 h, and co-stained with DAPI. The cellular localization of Flag tagged XPD protein was analyzed using a Leica confocal laser scanning microscope TCS SP (Leica).

### Sub-cellular fractionation and protease treatment or alkali extraction on isolated mitochondrial fraction

Isolation of mitochondria was performed using Qiagen Qproteome mitochondria isolation kit. Briefly, 1 × 10^7^ cells were resuspended in ice-cold Lysis buffer, incubated at 4°C for 10 min and then centrifuged at 1000 g for 10 min. After removing the supernatant containing cytoplasmic protein, the pellets were resuspended in ice-cold disruption buffer and homogenized by using a 26 g needle, centrifuged at 1000 g for 10 min and supernatant was collected. After final centrifuging at 6000 g for 10 min, the pellets containing mitochondrial fraction were retrieved.

For proteinase K treatment, purified mitochondria were resuspended in mitochondrial storage buffer and digested with proteinase K (100 ng/ml) on ice in the presence or absence of 1% TritonX-100 for 30 min. XPD expression was monitored by western blotting. For mitochondrial alkali extraction, mitochondria were treated with 0.1 M Na_2_CO_3_ (pH 11.5) for 30 min on ice, and then centrifuged at 16 000 g for 15 min. Supernatants (S) containing mitochondrial soluble and peripheral membrane proteins were retained. The pellet (P) containing mitochondrial membrane protein were washed once and then suspended in an equal volume of mitochondrial suspension medium I (20).

### Hydrogen peroxide (H_2_O_2_) treatments

HEK293 and U2OS cells in FBS-free DMEM medium as well as NHDF and XPD cells in FBS-free DMEM/F12 medium were treated with 0.5 or 1 mM H_2_O_2_ for 1 h at 37°C. After washing with PBS, media were replaced by complete culture medium and the cells were collected at the indicated time-points post treatment.

### Mitochondrial superoxide production

Both XPD-suppressed and scrambled shRNA-transfected U2OS cells were cultured on chamber slides for 48 h and then stained with 5 μM MitoSOX^TM^ Red (Molecular Probes) for 30 min at 37°C. After three gentle washes with pre-warmed PBS, cells were trypsinized and resuspended in BD Falcon tubes (BD Biosciences) at a concentration of 1×10^7^ cells/ml, and then analyzed using BD FACSAria (BD Biosciences). The fluorescence was measured using excitation at 510 nm and emission at 580 nm. For statistical analysis, at least 10 000 events were collected for each of three independent experiments. Data analysis was performed with winmdi28 software, and the mean fluorescence intensity (PE-A) was used to quantify the responses. Relative ROS production was calculated by mean values of PE-A from three independent experiments.

### Genomic DNA isolation and long-range quantitative PCR

Genomic DNA was isolated using Qiagen genomic DNA isolation kit (Qiagen). Measurement of mtDNA damage and repair was performed using quantitative PCR (QPCR) that amplify long mtDNA (16261 bp) target (21). A 13.5-kb product from *β-globin* gene was also used for measuring the nuclear DNA damage and repair. The mitochondrial NADH dehydrogenase 1 (*mtND1*) was first quantified by real-time PCR using SYBR Green detection on a CFX96 Real-Time PCR Detection System (Bio-Rad) using the following primers: Nu18S-F, 5′-TAGAGGGACAAGTGGCGTTC; Nu18S-R, 5-CGCTGAGCCAGTCAGTGT; mtND1–3212F, 5′-CACCCAAGAACAGGGTTTGT; mtND1–3319R, 5′-TGGCCATGGGTATGTTGTTAA (22). The input of mtDNA template was adjusted based on *mtND1* to ensure that equal amount of mtDNA template in each sample was used for the long-range QPCR reaction. The *mtND1* PCR product was used to normalize the data obtained with the 16.2-kb mtDNA fragment.

The primers used were the following: *β-globin* gene: sense: 5′-CGAGTAAGAGACCATTGTGGCAG and antisense: 5′-GCACTGGCTTAGGAGTTGGACT; mtDNA gene: sense: 5′-TGAGGCCAAATATCATTCTGAGGGGC and antisense: 5′-TTTCATCATGCGGAGATGTTGGATGG (23,24). PCR reactions were performed at 94°C for 1 min followed by 25 cycles at 98°C for 10 s, 60°C for 40 s, 68°C for 16 min and a final elongation for 10 min (14). The products were separated by electrophoresis on a 0.8% agarose gel stained with ethidium bromide. All reactions were done in triplicate and three independent experiments were performed. Relative mtDNA in each sample was calculated by Image J software.

### Measurement of common deletion

The cells were treated with 0.5 mM H_2_O_2_ with or without 5000 U/ml Catalase at 37°C for 1 h. At 0 h and 48 h time-point post treatment, the cells were collected and their genomic DNA was isolated as above. Measurement of common deletion (CD) was performed by real-time PCR using SYBR Green detection (Takara) on a CFX96 (Bio-Rad). The sequence spanning the CD was amplified using the primers: CD-F, 5′-ACCCCCATACTCCTTACACTATTCC and CD-R, 5′-AAGGTATTCCTGCTAATGCTAGGCT. The 83 bp fragment serving as internal standard for total mtDNA was amplified with primers: IS-F, 5′-GATTTGGGTACCACCCAAGTATTG and IS-R, 5′- AATATTCATGGTGGCTGGCAGTA (25,26).

### Co-immunoprecipitation assay and mitochondrial isolation

The large-scale mitochondrial fraction was isolated following the protocol reported previously (27). In brief, HEK293-Flag-HA-XPD cells from 40 of 150 mm tissue culture dishes were trypsinized and suspended in six volumes of ice-cold CHM buffer (cell homogenization medium, 150 mM MgCl_2_, 10 mM KCl, 10 mM Tris-HCl pH 6.7). After homogenizing using a 26 g needle syringe for ∼30 times, 1/3 volume of ice-cold CHM containing 1 M sucrose (final 0.25 M) was added, mixed gently by repeated inversion and centrifuged at 1000 g for 10 min at 4°C. The supernatant was collected and centrifuged at 5000 g for 10 min at 4°C. Finally, the pellet was suspended in ice-cold sucrose/Mg^2+^ medium (0.15 M MgCl_2_, 0.25 M sucrose, 10 mM Tris-HCl pH 6.7) and re-centrifuged at 5000 g for 10 min at 4°C. At last, purified mitochondria were resuspended in mitochondrial suspension medium I (0.25 M sucrose, 10 mM Tris-HCI pH 7.0), solubilized in lysis buffer (50 mM Tris-HCl pH 7.5, 150 mM NaCl, 1% NP-40, 5 mM EDTA, 5 mM EGTA, 20 mM NaF, 0.1 mM PMSF, 0.5 mM benzamidine, 1 mg/ml leupeptin, 1 mg/ml aprotinin, 2 mM microcystin and 0.1 mM NaVO3), and then incubated with anti-Flag-M2 beads (Sigma) at 4°C for 2 h. After washing with lysis buffer, the binding complex was eluted by Flag peptides, and heated at 80°C for 10 min in 1×Laemmli buffer. The XPD complex was visualized by sodium dodecyl sulphate-polyacrylamide gel electrophoresis followed by silver staining. The bands was collected and used for LC-MS/MS analysis. The physical interaction between XPD and candidates was further validated by Co-IP (28).

## RESULTS

### XPD protein is localized to mitochondria

**Figure 1.**
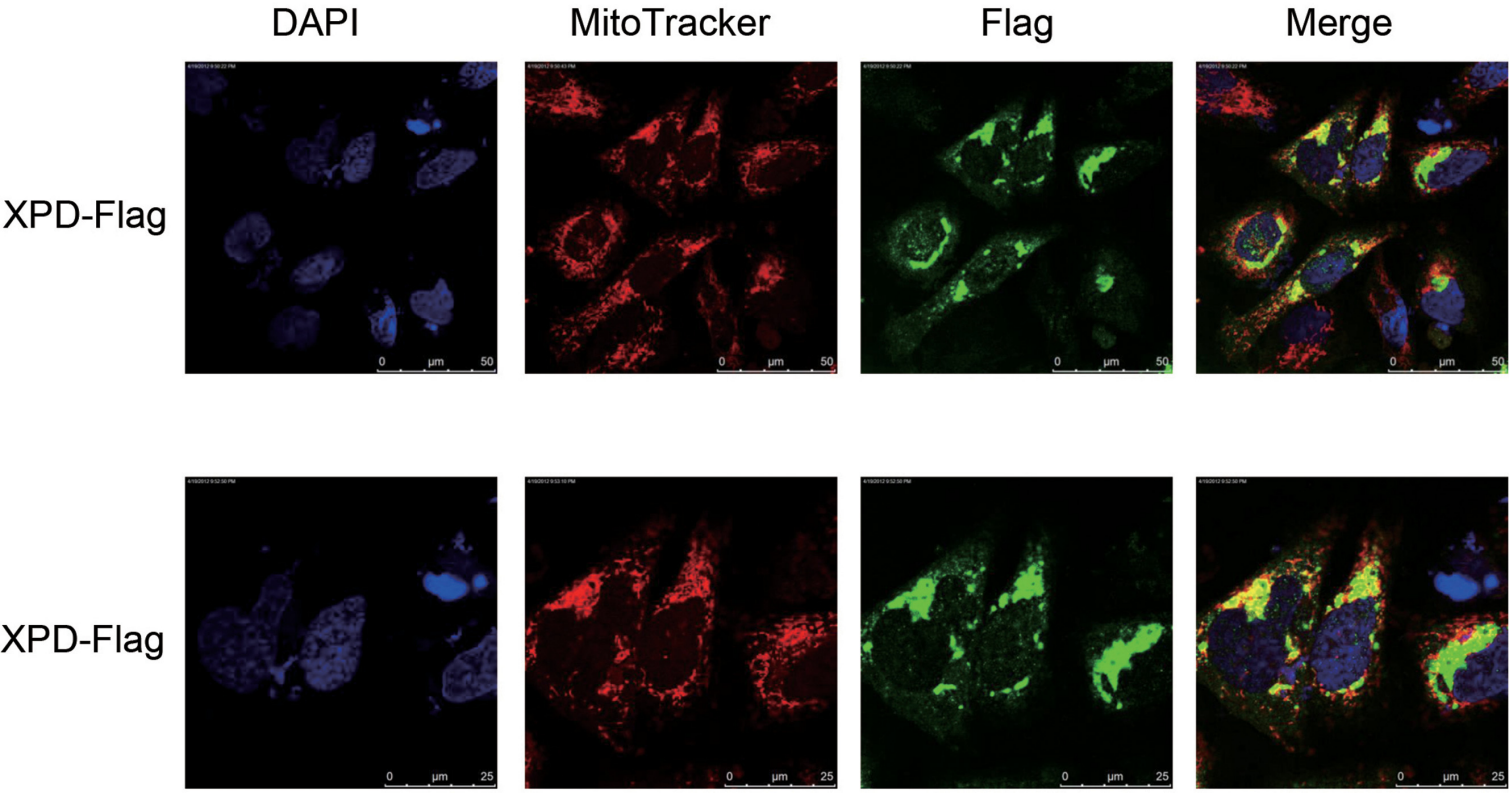
Co-localization of XPD protein with mitochondria in U2OS cells by an indirect immunofluorescence approach. The cells were transfected with pcDNA3.0-Flag-XPD expression plasmid, and stained by 5 μM of Mitotracker Red CM-H2Xros for 30 min at 24 h post transfection. After fixation in 4% paraformaldehyde for 10 min and 0.1% Triton X-100 for 5 min, the cells were incubated with the first Flag antibody (Sigma) overnight at 4°C followed by fluorescein goat anti-mouse IgG secondary antibody (Vector laboratories) at RT for 2 h. The staining result was visualized under the Leica Confocal microscope.

**Figure 2.**
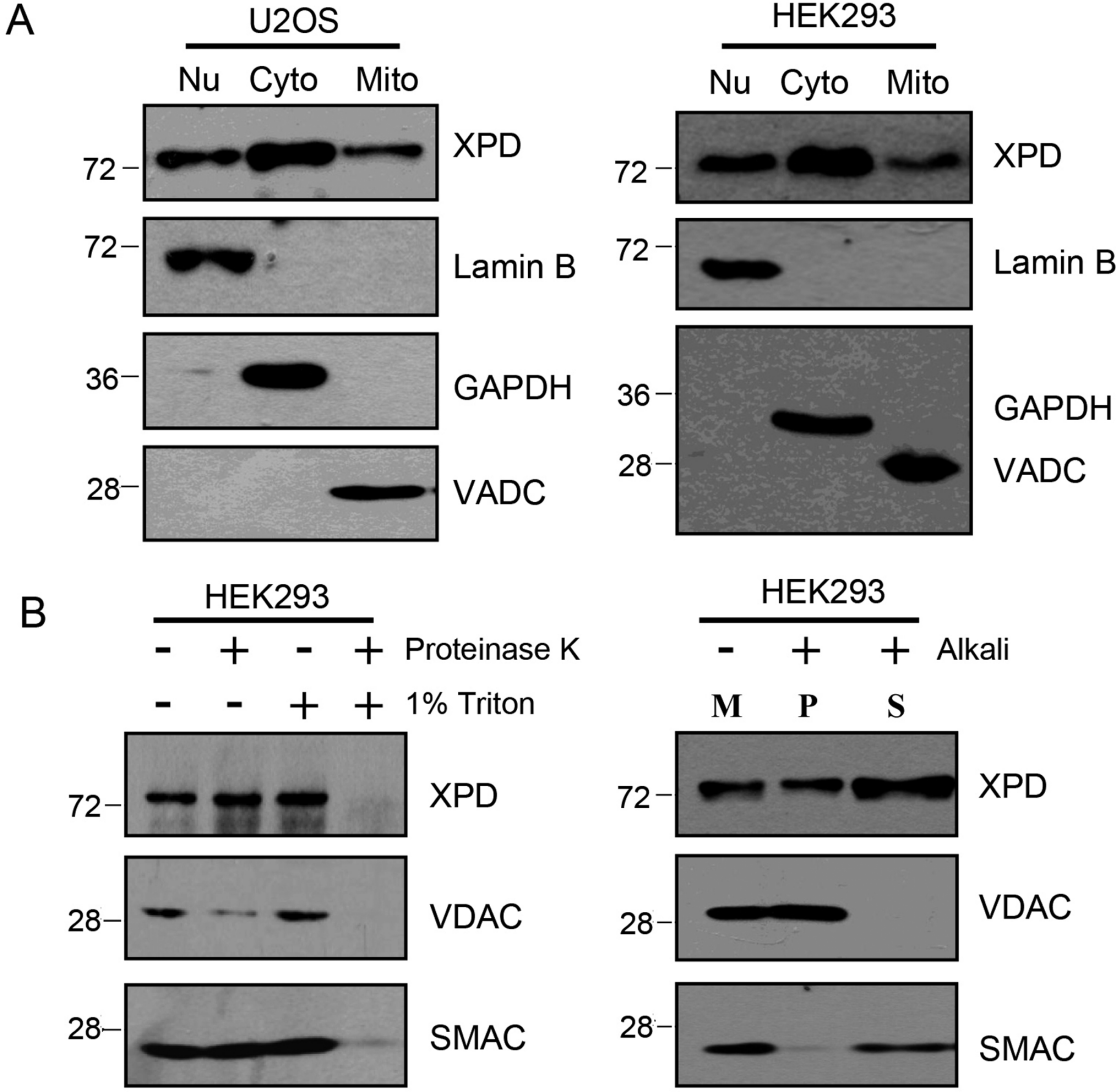
Localization of XPD in mitochondria examined by western blotting coupled with protease treatment or alkali extraction on isolated mitochondrial fraction. (**A**) Endogenous level of XPD protein in both nucleic and mitochondrial fractions of U2OS and HEK293 cells determined by western blotting. Presence of XPD protein in mitochondrial fraction of both cell types was observed. XPD antibody was purchased from Cell Signaling. Anti-VDAC (mitochondrial marker), Lamin B (nucleic marker) and GAPDH (cytoplasmic marker) were ordered from Cell Signaling, Santa Cruz, Millipore, respectively. (**B**) Protease treatment was performed on intact mitochondria fraction isolated from HEK293 cells by digestion with 100 ng/ml proteinase K (PK) in the presence or absence of 1% TritonX-100 on ice followed by western blotting analysis. VDAC serves as the marker of mitochondrial outer membrane protein, and SMAC as the marker of mitochondrial intermembrane space protein. Anti-SMAC antibody was purchased from Cell Signaling. Alkali extraction was carried out by using Na_2_CO_3_ to treat the mitochondrial fraction isolated from HEK293 cells. Both the soluble protein fraction (S) and integral membrane protein fraction (P) were isolated and used for western blotting analysis. ‘M’ means untreated control mitochondrial sample. Results demonstrate the localization of XPD in the mitochondrial inner matrix compartment and inner mitochondrial membrane.

Cytoplasmic and nuclear localization of XPD have been demonstrated earlier and that XPD participates in NER of nuclear DNA (3). As mitochondria are constantly challenged by oxidative stress, we hypothesized that XPD protein may be localized in mitochondria for repairing oxidative DNA damage in mtDNA. In corroboration with an earlier report (29), our immunofluorescent studies demonstrated the cytoplasmic and nuclear distribution of XPD with a predominant portion residing in the cytoplasm. Strikingly, co-localization of overexpressed XPD protein and mitochondria was clearly observed in pcDNA3.0-Flag-XPD transfected U2OS cells (Figure 1). Western blotting analysis further demonstrated the presence of XPD protein in the mitochondrial fractions isolated from U2OS and HEK293 cells. In corroboration with the immunofluorescence studies, mitochondrial fraction exhibited a much lower level of XPD protein relative to cytoplasm/nuclear fractions. Lack of detectable levels of nuclear protein marker Lamin B and cytoplasmic protein marker GAPDH indicated that the mitochondrial fraction was largely free of nuclear and cytoplasmic contaminations (Figure 2A).

Protease protection assays were next performed to further demonstrate the location of XPD protein within the mitochondria. The results showed that when mitochondria were treated with 100 ng/ml proteinase K in the absence of 1%Triton X-100 for 30 min on ice, most of VDAC, the outer mitochondrial membrane protein, was digested, whereas both SMAC, the mitochondrial intermembrane space protein, and XPD were resistant to protease digestion indicating that XPD is located inside of the mitochondria (Figure 2B). When mitochondria were subjected to alkali extraction, peripheral membrane proteins were recovered in the supernatant, while integral membrane proteins were found in the membrane-containing pellet fractions. As expected, the outer membrane integral protein VDAC was primarily recovered in the pellet following extraction while the intermembrane space protein SMAC was largely recovered in the supernatant. XPD was observed in both fractions, indicating that XPD is located not only in the integral membrane, but partially in the soluble fraction as well.

### XPD silencing elevates ROS production in mitochondria

**Figure 3.**
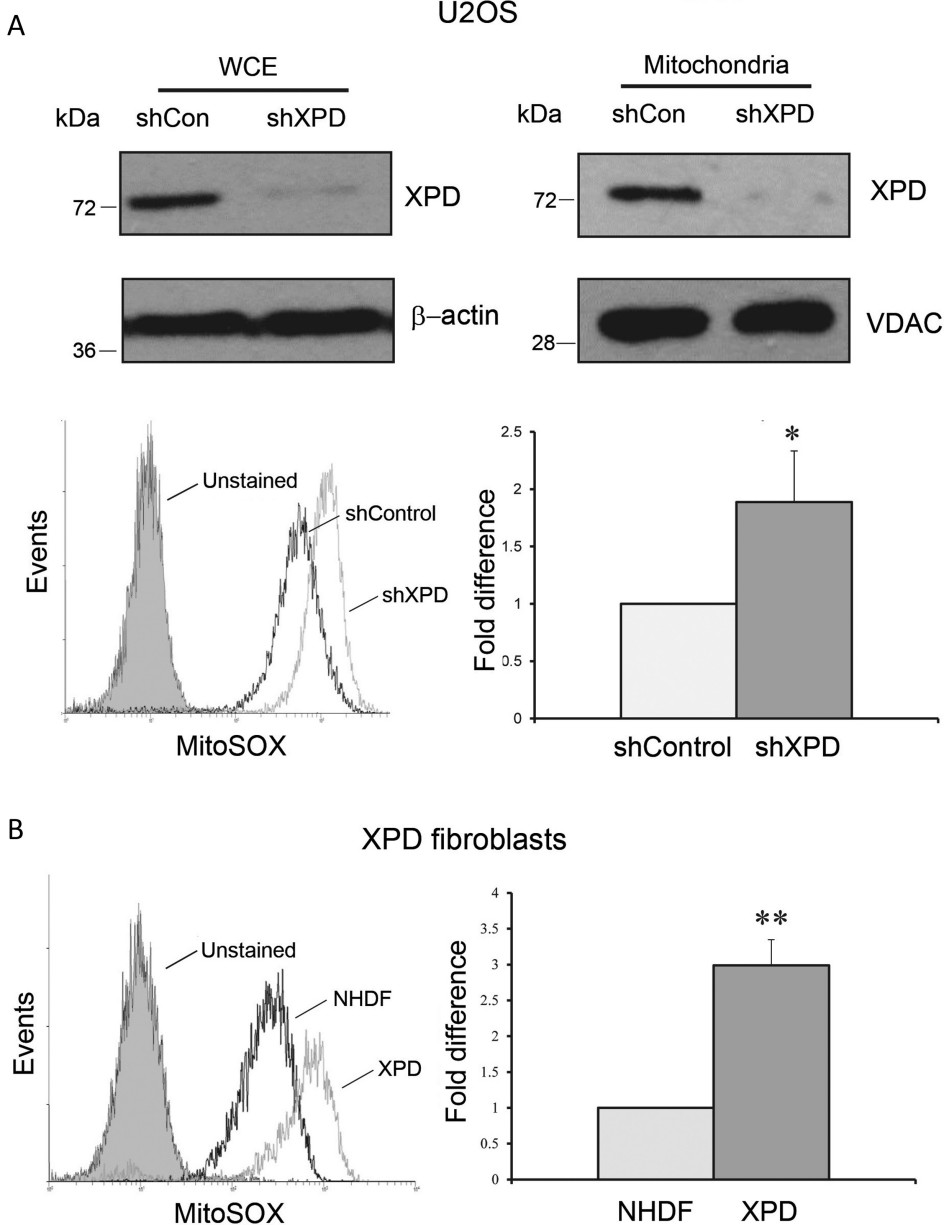
Mitochondrial ROS production in XPD silenced U2OS and XPD patient skin fibroblasts. (**A**) XPD levels in whole cell extracts (WCE) and mitochondrial fraction of U2OS determined by western blotting. A markedly decreased level of XPD was observed in both samples. Mitochondrial ROS level in shControl and shXPD U2OS cells was detected by MitoSOX^TM^ Red. *P < 0.05. (**B**) Mitochondrial ROS level in normal human dermal fibroblasts (NHDF) and *XPD*-deficient patient skin fibroblasts. Cells were stained with MitoSOX^TM^ Red for 30 min and then analyzed by flow cytometry. Relative ROS production was calculated by PE-A means from three independent experiments. Error bars represent standard deviations. **P < 0.01.

Co-localization of XPD protein and mitochondria led us to verify whether or not XPD plays a role in repairing mtDNA damages as it does in the nucleus. Mitochondrial oxidative stress was assessed in XPD-suppressed U2OS cells using MitoSOX^TM^ Red (30). Both scrambled control and XPD shRNA transfected cells were stained with MitoSOX^TM^ Red for 30 min and subsequently analyzed by flow cytometry. As shown in Figure 3A, the curve formed by MitoSOX^TM^ Red-positive cells after XPD suppression shifted markedly to the right of shControl cells, indicating an elevated level of oxidative stress in U2OS cells after XPD silencing. The ROS production in XPD-suppressed cells was over 2-fold higher than in shControl cells.

We next determined whether or not human fibroblasts that are inherently deficient in XPD also exhibit an increased ROS production. For this purpose, XPD-deficient human fibroblasts (GM00434) and control NHDF human normal dermal fibroblasts were compared for their ROS levels. Consistent with our observation in U2OS cells, XPD-deficient human fibroblasts also displayed an increased mitochondrial ROS production with ∼3-fold higher than normal human fibroblasts (Figure 3B).

### XPD protein level was increased in mitochondria under oxidative stress

**Figure 4.**
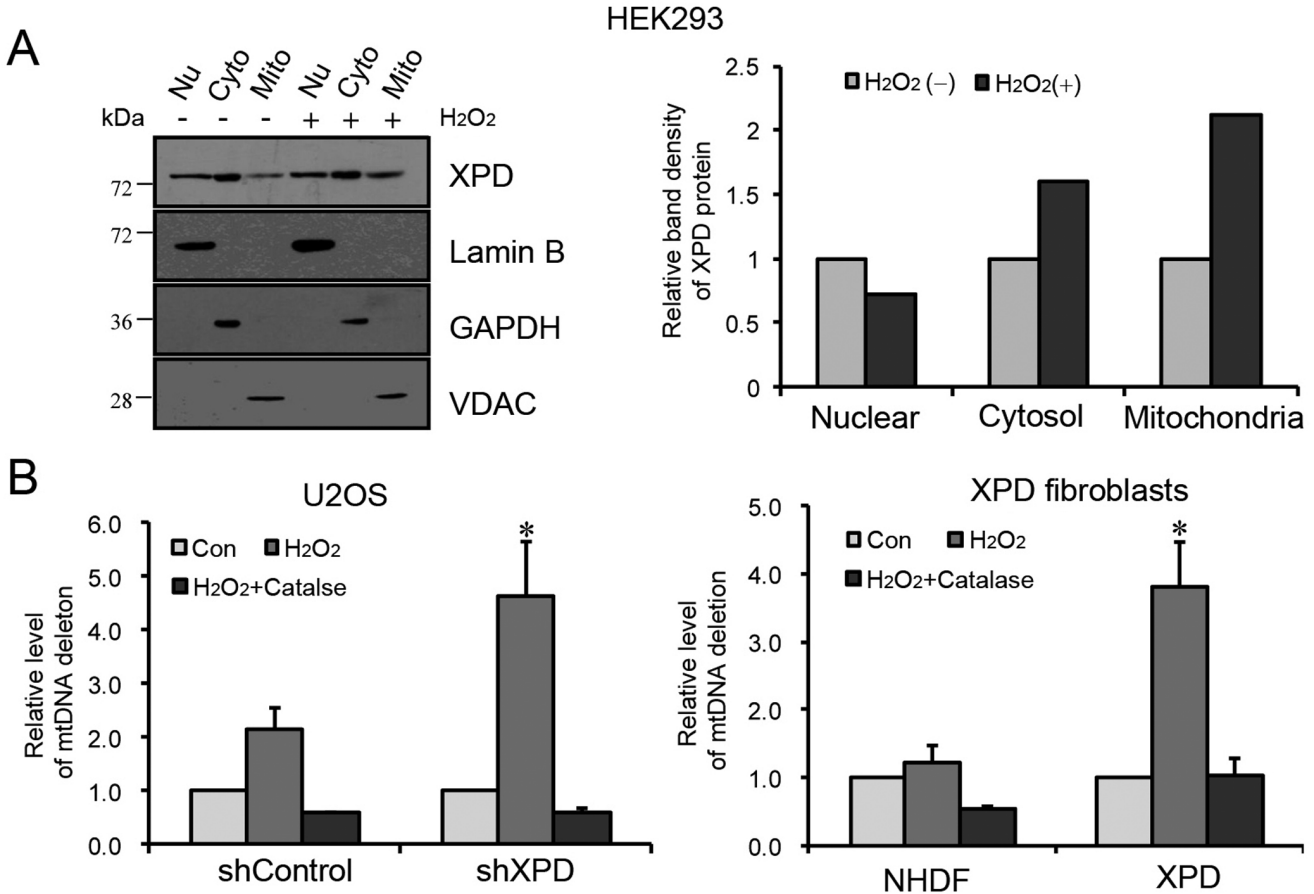
Level of mtDNA CD post oxidative stress in XPD-wild type and deficient cells. (**A**) An increased mitochondrial distribution of XPD protein in HEK293 cells after H_2_O_2_ treatment. Cells were treated with 0.5 mM H_2_O_2_ for 1 h, and samples were collected at 45 min post treatment. Fifty micrograms of protein for each of nuclear, cytosolic and mitochondrial fractions were loaded and separated on SDS-polyacrylamide gel for western blotting analysis. Mitochondrial proportion of XPD protein was quantified using the Image J software (http://rsbweb.nih.gov/ij/) and normalized to mitochondrial loading control (VDAC). (**B**) The relative quantities of CD were measured by real-time PCR in XPD knock-down U2OS cells, normal NHDF versus XPD patient cells at 48 h post 0.5 mM of H_2_O_2_ treatment in the presence or absence of 5000 U/ml of Catalase. Levels of CD were assessed by Bio-Rad CFX Manager 2.1. Samples were collected at 0 h (Con) and 48 h recovery with or without catalase (H_2_O_2_ or H_2_O_2_+catalase) post treatment. Data were from three independent experiments. Error bars represent standard deviations. *P < 0.05.

We then tested whether or not XPD protein is enriched in mitochondria when the cells are challenged by oxidative stress. HEK293 cells were exposed to 0.5 mM H_2_O_2_ for 60 min in serum-free DMEM, then washed with PBS and cultured in full growth medium. After 45 min recovery, the cell lysates were prepared for the western blotting analysis (Figure 4A). In contrast to the untreated cells, XPD protein level was substantially increased in mitochondria under oxidative stress condition with 2-fold increase relative to controls.

### XPD deficiency leads to a higher level of mtDNA CD

Oxidative stress can cause the accumulation of point mutations and deletions in both genomic and mitochondrial DNA. The most frequently reported mtDNA mutation is a specific 4977-bp deletion, referred to as CD, that had been used as a marker for measuring oxidative stress-induced mtDNA mutations (25,31,32). The potential effect of XPD protein in protecting the mtDNA from oxidative stress damage was examined by quantitative real-time PCR (26). As shown in Figure 4B, XPD-suppressed (shXPD) and control (shControl) cells were treated with 0.5 mM H_2_O_2_ for 60 min and allowed to recover for 48 h. In contrast to shControl cells, shXPD cells showed >2-fold higher induction of CD, suggesting that XPD is essential for protecting mtDNA from CD mutations arising from exogenous oxidative DNA damage. In support, XPD-deficient human fibroblasts also showed a 3-fold higher level of CD than NHDF cells after treatment with 0.5 mM H_2_O_2_ for 60 min followed by a recovery time of 48 h. Specifically, addition of 5000 U/ml natural antioxidant catalase concurrently with H_2_O_2_ treatment decreased the CD level in XPD-silenced U2OS cells and *XPD*-deficient human fibroblasts to the level of controls, suggesting that XPD deficiency could lead to an increased oxidative mtDNA damage that can be overcome by catalase, an antioxidant agent.

### XPD deficiency results in a defective mtDNA repair

**Figure 5.**
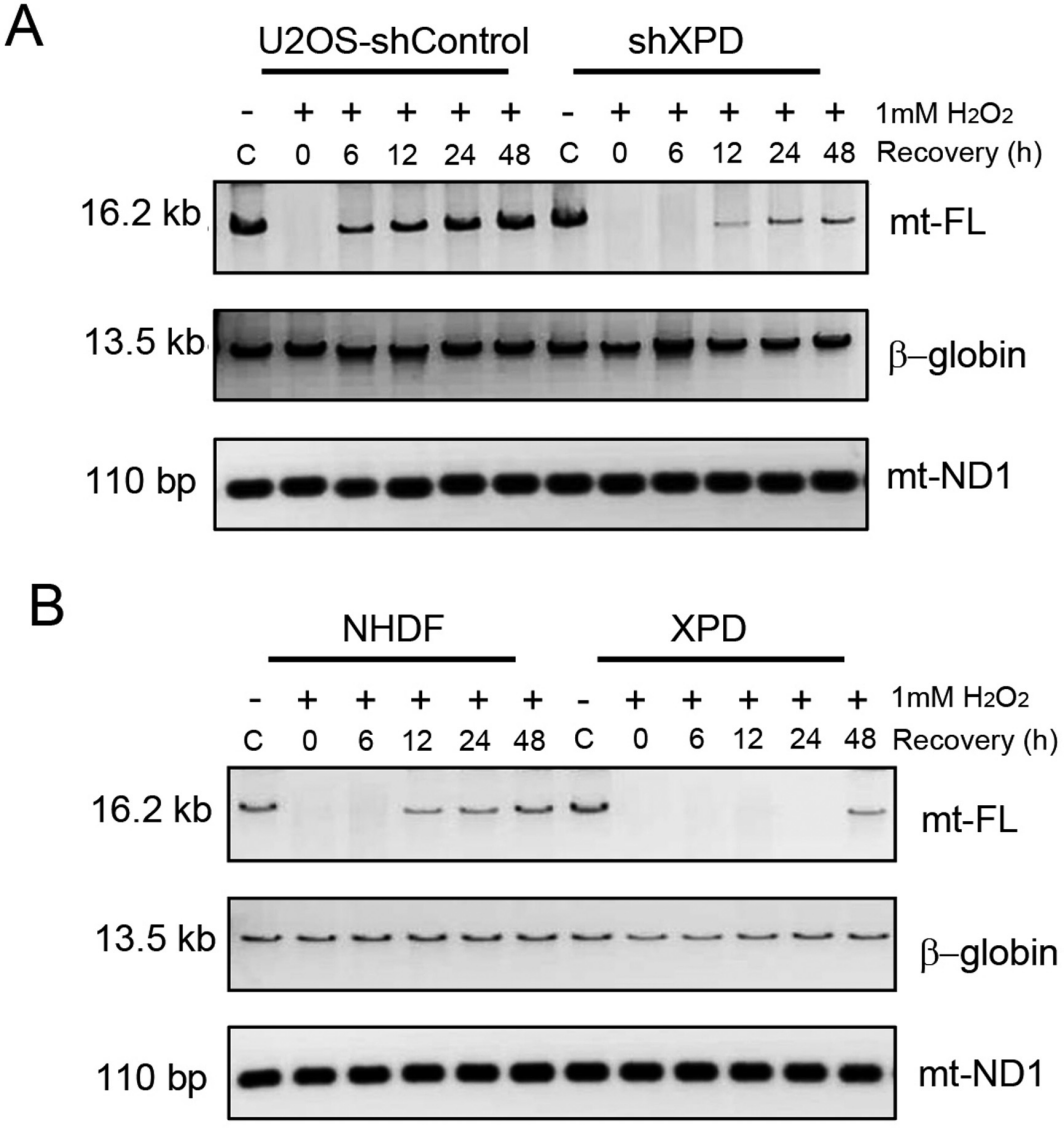
Long-range QPCR results of full-length mitochondrial DNA (mt-FL) in shControl and shXPD U2OS cells (**A**) and in NHDF and XPD patient fibroblasts (**B**) at recovery time-points of 0, 6, 12, 24 and 48 h post 1 mM H_2_O_2_ treatment for 1 h. Equal amount of mtDNA template in each sample normalized by *mtND1* gene was used for long-range QPCR reactions. Nucleic *β-globin* gene was used as the repair efficiency marker for oxidative nuclear DNA damage.

A long-range QPCR approach for amplification of 16.2 kb mtDNA was subsequently used to assess the repair efficiency of mtDNA (23). A major advantage of this method is that any damaged and unrepaired bases will terminate the polymerase elongation during PCR amplification thereby reducing the efficiency of DNA amplification. The mtND1 PCR product (110 bp) was used as a control to normalize the input of the mtDNA template amount in each sample. The findings from 0.5 mM H_2_O_2_ treatment showed that XPD-deficient cells exhibited a higher level of initial oxidative damage (0 h post treatment) and a much attenuated repair capacity in mitochondria in response to oxidative stress in relative to XPD-proficient cells (Supplementary Figure S1). To further determine whether or not repair kinetics differs between XPD-proficient and deficient cells, additional experiments were performed by treating the cells with 1 mM concentration of H_2_O_2_. As shown in Figure 5A, both shControl and XPD knock-down U2OS cells showed a markedly decreased level of mtDNA PCR product at early time-points after H_2_O_2_ treatment. In contrast to control cells that had a marked restoration of full-length mtDNA PCR product by 6 h and complete recovery by 24 h post treatment, XPD silenced cells showed a very low level of PCR product even at 48 h post treatment. Consistently, XPD-deficient fibroblasts also displayed a much delayed mtDNA repair post H_2_O_2_ treatment as compared to NHDF cells (Figure 5B). The quantification data was included in Supplementary Figure S2A. These findings clearly illustrate that XPD is essential for the efficient repair of oxidative DNA damage in mitochondria. However, amplification of nuclear *β-globin* gene was not affected after hydrogen peroxide treatment either in XPD-suppressed U2OS cells or in *XPD* mutated human fibroblasts relative to untreated control cells, illustrating that XPD is an essential DNA repair factor for oxidative damage in mitochondria but is dispensable for some of the nuclear genes such as *β-globin*.

### XPD helicase activity is critical in XPD-regulated mtDNA repair

**Figure 6.**
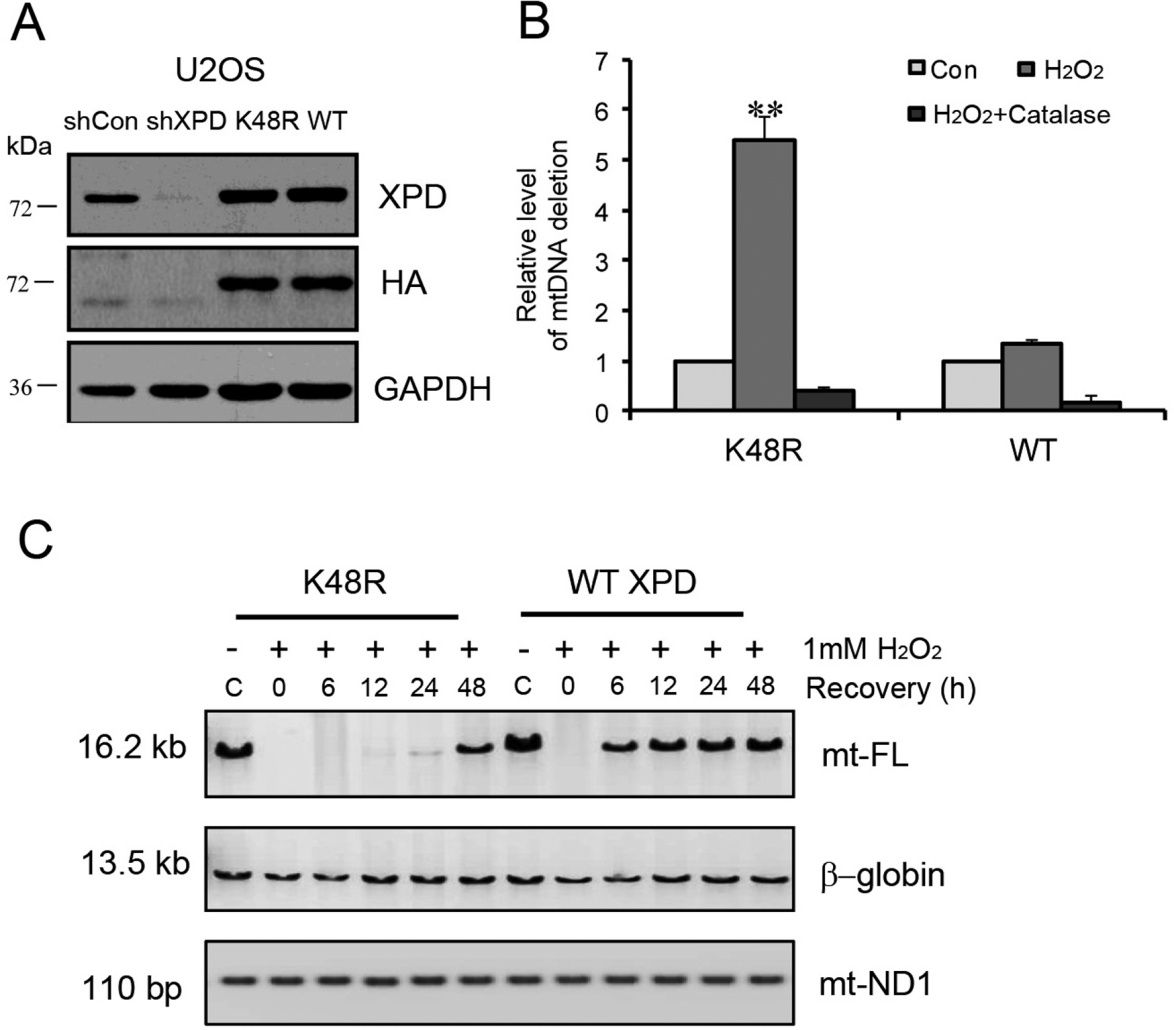
XPD helicase activity is critical in XPD-regulated mtDNA repair. (**A**) Reconstitution of HA-WT XPD and HA-XPD/K48R mutant in XPD-silenced U2OS cells examined by western blotting. (**B**) Level of CD in XPD-WT or mutant reconstituted XPD-silenced U2OS cells quantified by real-time PCR at 0 h (Con), 48 h (H_2_O_2_ or H_2_O_2_+catalase) post H_2_O_2_ treatment. Cells were treated with 0.5 mM H_2_O_2_ for 1 h. Data from three independent experiments. (**C**) Long-range QPCR results of full-length mitochondrial DNA (mt-FL) in XPD-WT versus helicase mutant-reconstituted XPD-silenced U2OS cells at recovery time-points of 0, 6, 12, 24 and 48 h post H_2_O_2_ treatment.

It has been reported that XPD helicase activity is exclusively devoted to DNA repair (19). To determine the role of XPD helicase activity in mtDNA repair, reconstitution assay and functional analysis were performed on XPD knock-down U2OS cells by transfection with pcDNA3.1-HA-WT/XPD and pcDNA3.1-HA-XPD/K48R expression plasmid, respectively. After confirming the expression levels of wild-type XPD and XPD helicase mutant proteins by western blotting using anti-XPD and anti-HA antibodies (Figure 6A), functional assays (CD assay and long-range QPCR analysis) were subsequently performed. As shown in Figure 6B, XPD knock-down cells with XPD/K48R reconstitution still showed a significantly higher level of CD after challenging with H_2_O_2_, whereas its level was markedly decreased in WT-XPD reconstituted cells. Moreover, the repair capacity for mtDNA oxidative damage was mostly recovered after complement of WT-XPD instead of XPD/K48R mutant in XPD-silenced cells (Figure 6C, Quantification data in Supplementary Figure S2B), suggesting an essential role for XPD helicase activity in mtDNA oxidative damage repair.

### Purification of XPD protein complex in mitochondria

**Figure 7.**
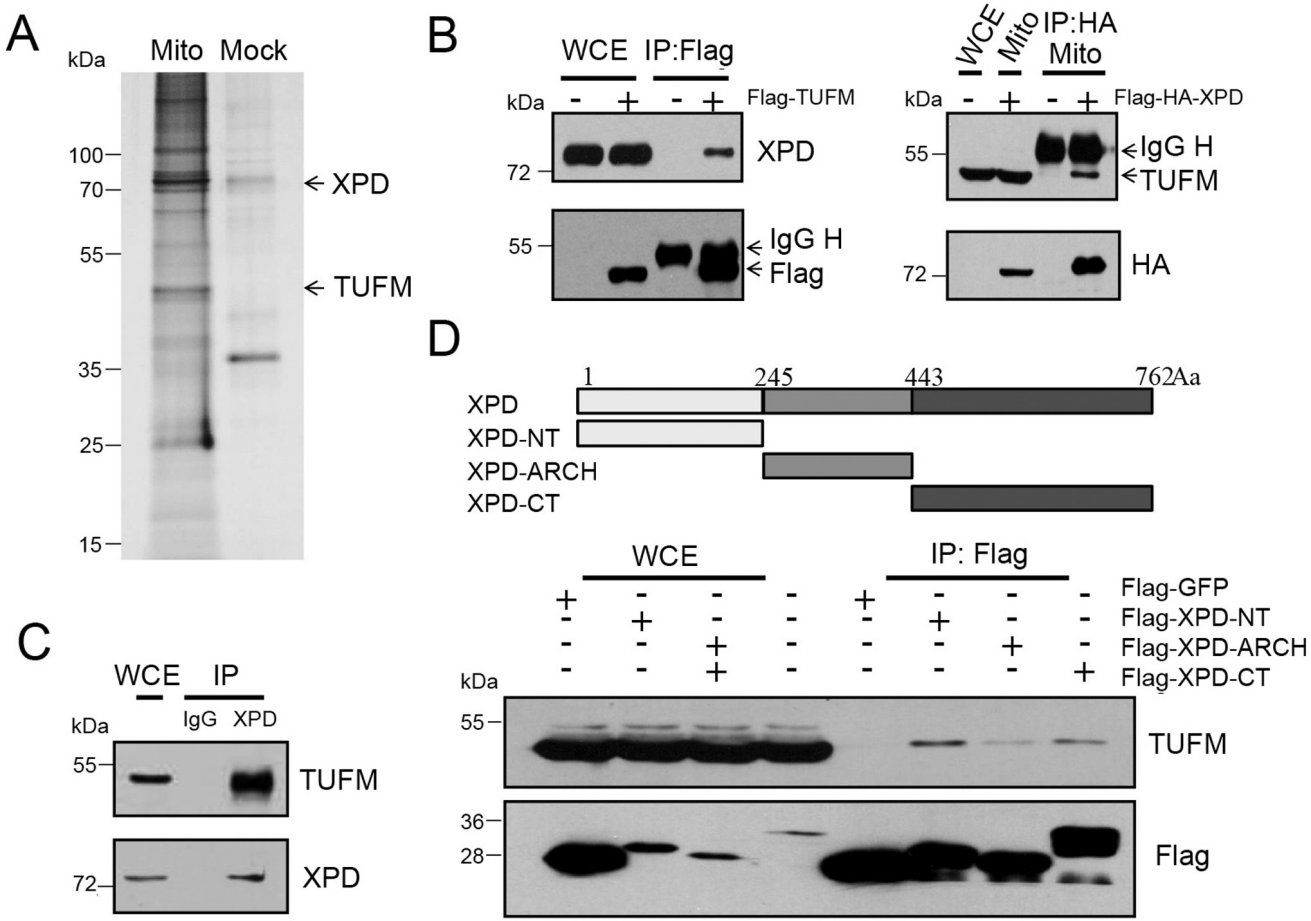
XPD associates with mitochondrial TUFM. (**A**) Silver-staining of XPD-associated proteins. Mitochondrial fraction isolated from HEK293 cells stably expressing Flag-XPD were immunoprecipitated with antibody against Flag M2 beads and analyzed by a Mass-Spectrometer (lane 1). Lane 2 is mock control without transfection with Flag-XPD expression vector. TUFM (arrow) was identified to be one of the partners of XPD. (**B**) Endogenous XPD was immunoprecipitated with Flag-TUFM recognized by an anti-Flag antibody from cell extracts of 10^6^ HEK293 cells. The immunoprecipitated proteins were detected with antibody against XPD (Cell Signaling). Conversely, endogenous TUFM was immunoprecipitated with Flag-XPD recognized by an anti-Flag antibody from mitochondrial extracts of 10^6^ HEK293 cells. The immunoprecipitated proteins were visualized by western blotting analysis with antibody against TUFM (Sigma). Five percent of the lysate was used for the loading control (Input) and the remaining 95% for co-IP. (**C**) Endogenous TUFM was immunoprecipitated with anti-XPD antibody (Cell Signaling) from cell extracts of 10^6^ HEK293 cells. TUFM in the pull-down complex was visualized by western blotting analysis using antibody against TUFM (Sigma). (**D**) In the upper panel, schematic diagram of XPD deletion constructs used for co-IP studies is shown. In the lower panel, HEK293 cells were transfected with either Flag-GFP or one of the Flag-tagged truncated XPD expressing plasmids. TUFM was strongly immunoprecipitated with XPD N-terminal domain, while weakly with XPD C-terminal (CT) and ARCH domains.

XPD is involved in NER pathway in the nuclear genome and XPD is an integral component of TFIIH, a nine sub-unit complex, that plays dual roles in both RNA polymerase II transcription and NER (3). Since XPD interacts with TFIIH sub-units in NER pathway, it is likely that XPD may cooperate with other factors in the repair of oxidative DNA damage specifically in mitochondria. To identify and characterize the potential factors that may interact with XPD in mitochondria, immunoprecipitation combined with mass spectrometry analysis was performed on mitochondrial protein fraction. Using this approach, we identified TUFM, a mitochondrial Tu translation elongation factor, as one of the interacting factors for XPD (Figure 7A). To further validate this observation, co-immunoprecipitation (co-IP) assay was performed. For this purpose, whole cell lysate from HEK293 cells expressing Flag-TUFM was used. When Flag-TUFM was precipitated with anti-Flag antibody, endogenous XPD was detected by anti-XPD antibody, demonstrating the physical interaction between TUFM and XPD (Figure 7B). Likewise, endogenous TUFM was detected in mitochondrial lysate immunoprecipitated with anti-HA antibody in HEK293 cells expressing Flag-HA-XPD. To exclude the possible artifact due to Flag-XPD expression, co-IP analysis was performed using anti-XPD antibody to immunoprecipitate endogenous TUFM from the lysate of HEK293 cells. As shown in Figure 7C, endogenous TUFM was observed in the pull-down complex, illustrating the physical interaction between XPD and TUFM.

To determine which of the XPD functional domains interacts with TUFM, various truncated *XPD* constructs with only N-terminal (NT), C-terminal (CT) or ARCH domain were expressed in HEK293 cells and immunoprecipitated with anti-Flag antibody. Endogenous TUFM in the pull-down lysate was then detected using anti-TUFM antibody. The strongest interaction was observed in *XPD*-NT. The weak interaction was found in *XPD*-CT, then *XPD*-ARCH with TUFM (Figure 7D), suggesting that the interaction of XPD with TUFM is mediated mainly through NT of XPD protein.

### TUFM plays a similar role in mitochondrial oxidative damage repair as XPD

**Figure 8.**
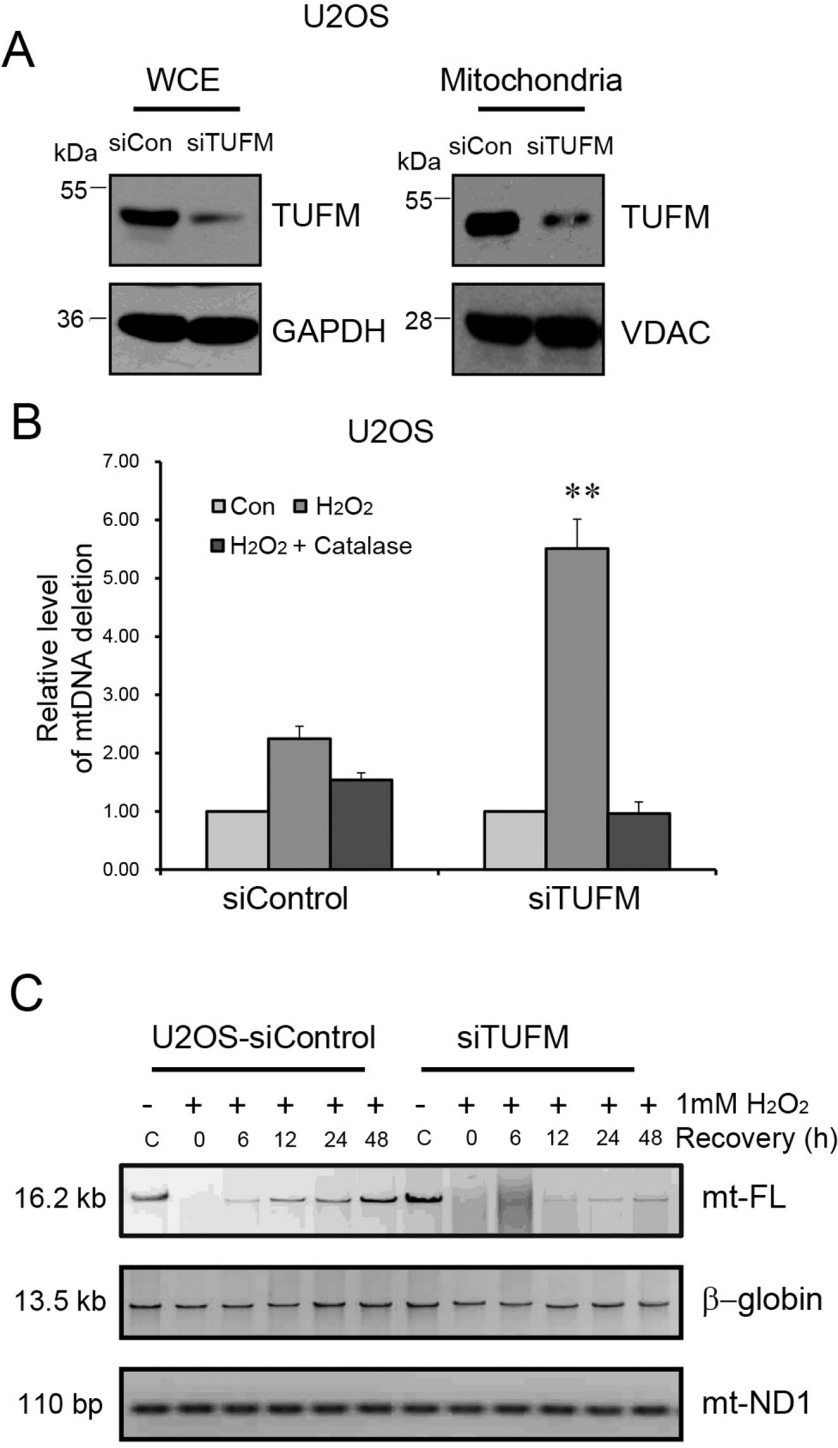
CD and oxidative damage repair in mitochondria after TUFM knock-down. (**A**) U2OS cells were transfected with control siRNA (siControl) and *TUFM* siRNA (siTUFM), respectively. Levels of TUFM in whole cell lysate and mitochondrial fraction were determined by western blotting. (**B**) CD of mtDNA was quantified by real-time PCR in siControl and siTUFM transfected U2OS cells. Cells were treated with H_2_O_2_ for 1 h in the presence or absence of catalase, and collected at 0 h and 48 h post treatment. Three independent experiments were carried out. Error bars represent standard deviations. **P < 0.01. (**C**) Result of long-range QPCR result of full-length mitochondrial DNA in siControl and siTUFM U2OS cells treated with 1 mM H_2_O_2_ for 1 h. Equal loading of DNA was verified by monitoring the level of mt-ND1 gene.

We next determined whether or not TUFM is involved in oxidative mtDNA damage repair. Control and *TUFM* siRNA were transfected into the U2OS cells, respectively. A marked reduction of TUFM expression was clearly observed in *TUFM* siRNA-transfected cells by western blotting (Figure 8A). TUFM suppression, unlike XPD suppression/deficiency, did not result in a marked shift for the curve formed by MitoSOX^TM^ Red-positive cells (data not shown). However, a significantly increased level of mitochondrial CD, similar to XPD-suppressed cells, was observed in TUFM suppressed cells post treatment with 0.5 mM H_2_O_2_ (Figure 8B). Additionally, the capacity for mtDNA oxidative damage repair quantified by long-range mtDNA PCR was also substantially decreased after TUFM reduction in U2OS cells when exposed to 0.5 mM H_2_O_2_ (Supplementary Figure S3).

To explore the molecular crosstalk between XPD and TUFM in mitochondrial oxidative DNA repair process, kinetics of mitochondrial DNA repair was assayed in TUFM knock-down cells after exposure to a higher concentration of H_2_O_2_ (1 mM). Strikingly, both siControl and TUFM knock-down U2OS cells had a markedly decreased level of mtDNA PCR product at early time-points after H_2_O_2_ treatment. However, control cells showed the restoration of full-length mtDNA PCR product by 48 h, whereas TUFM silenced cells displayed a very low level of PCR product even at 48 h post treatment (Figure 8C). Therefore, similar as XPD, TUFM deficiency also led to a much attenuated repair kinetics for mtDNA oxidative damage relative to wild-type cells. The quantification data was included in Supplementary Figure S2C. These findings clearly suggest that the interaction between XPD and TUFM is critical for an efficient formation of oxidative DNA damage repair machinery in mitochondria. Observations of diminished repair capacity in both XPD and TUFM suppressed cells lend credence to our suggestion.

## DISCUSSION

Mitochondria, as the ‘energy powerhouse of the cells’, participates in the regulation of cellular metabolism, cell-cycle control, development, antiviral responses and cell death (33). Mutations of mtDNA can cause a variety of diseases characterized by deafness, cancer predisposition cardiomyopathy and neurodegenerative disorders (34). Since mitochondria is devoid of the protective histone protein and is constantly challenged by free radical species causing mtDNA double-strand breaks or DNA adducts, an efficient DNA damage repair system is definitely required for maintaining mitochondrial genome stability. Both mismatch repair (MMR) and BER pathways have been shown to be actively take place in both the nuclear and mitochondrial genomes (35). However, there is no definite evidence showing the presence of NER pathway in the mitochondria although nuclear DNA adducts can be processed by NER (36). Recent reports demonstrated that several NER-associated proteins play an important role in oxidative mtDNA damage repair (26,37). Oxidative DNA damage induction is demonstrated to be several folds higher in mtDNA relative to nuclear DNA and the increased induction is probably owing to increased ROS generation in mitochondria. A recent report demonstrated that *XPD*-deficient lymphoblastoid cells are extremely sensitive to hydrogen peroxide induced oxidative DNA damage. Since XPD protein serves as an essential component of NER pathway and *XPD-*deficient cells are hypersensitive to oxidative DNA damages, a likely possibility is that XPD may also participate in the repair of oxidative DNA damage in both nuclear and mitochondrial genomes. However, it remains to be elucidated as to whether or not XPD localizes in mitochondria and protects the mitochondrial genome from oxidative DNA damage.

Available data from immunofluorescent staining demonstrated that XPD protein was mainly localized in the cytoplasm, with a minor fraction in the nuclear (38). XPD plays a different role based on its sub-cellular distributions. In nuclear, XPD participates in forming TFIIH complex by interacting with p44 and MAT1, which also contains XPB, p62, p52, p34, p8, cdk7 and cyclin H (39), and mainly functions in maintaining the stability of transcriptional complex during transcription and opening site of DNA damage by helicase activity in NER. In cytoplasm, XPD interacts with MMS19 forming MMXD complex mainly functioning in mitotic spindle formation and chromosome segregation which is thought to be independent of TFIIH (40). However, whether or not XPD protein resides and functions in mitochondria is still an enigma. Our immunofluorescent staining data showed that XPD was present in both the nuclear and cytoplasmic compartments with majority of the protein in the cytoplasm, which is consistent with the previous reports (29). More importantly, we observed that partial cytoplasmic XPD protein was co-localized with mitochondria, which was further confirmed by western blotting analysis on fractionated cell lysate showing the presence of the XPD protein in the mitochondrial fraction. In a likely possibility of artifactual proteins binding to mitochondria after breakup of the cells, one would expect their binding only to the outer membrane of mitochondria and such proteins can be easily digested by proteinase K. On the contrary, we observed that XPD can only be digested by proteinase K in the presence of a strong detergent such as Triton X-100. Further, alkali extraction assay demonstrated that XPD was present in both the membrane-containing pellet and soluble fraction of mitochondria. The XPD patterns from these two assays were found similar as the mitochondrial intermembrane space protein SMAC, suggestive of XPD localization in mitochondria. Further experiments are certainly warranted to elucidate the precise location of XPD within mitochondria.

The mtDNA is constantly challenged by the endogenous ROS and therefore an optimal level of DNA repair proteins is required in mitochondria for maintaining mtDNA stability. However, when mitochondria are challenged by an excess generation of ROS by exogenous agents such as H_2_O_2_, elevated level of DNA repair proteins such as XPD is required in mitochondria to remove the oxidative DNA damage efficiently from mtDNA. This assumption is fairly supported by our observations of XPD localization in mitochondria and its specific enrichment after oxidative stress in mitochondria. Our findings suggest that XPD may be a crucial repair factor for oxidative DNA damage in mitochondria. In support, our functional analyses demonstrated that XPD knock-down led to a significantly increased level of CD, and also a substantially lower capacity for repairing the oxidative mtDNA damage. It is well accepted that the accumulation of mutations and deletions in mtDNA can impair its function in the respiratory chain thereby enhancing the ROS production. Elevated ROS production in turn can subsequently lead to a vicious cycle of exponentially increasing the levels of mtDNA damage and oxidative stress in the cell (41). In corroboration, we have observed a significantly higher level of mtDNA CD and ROS production in both XPD knock-down U2OS cells and XPD-deficient human skin fibroblasts. Although the precise role of XPD in redox homeostasis remains to be elucidated, our study indicates that XPD deficiency is likely to result in defective oxidative damage repair of mtDNA leading to a higher degree of mtDNA mutations and consequently, an increased level of ROS production. Similar to our observations, defects in mitochondrial DNA repair as well as elevated ROS generation have been documented in many human and mouse cell lines with mutations in DNA repair genes (14,15,26,41). XPD serves as a critical factor in NER pathway (3), whose deficiency has been found to render the cells hypersensitive to oxidative stress (10,18,42). The fact that XPD deficiency selectively affects the oxidative DNA damage repair capacity of mtDNA but not nuclear genes such as *β-globin* suggests that XPD is a critical factor for oxidative DNA damage repair machinery operating in mitochondria (11). Therefore, it is tempting to speculate that the hypersensitivity of XPD-deficient cells to exogenous oxidative DNA damage is partly due to diminished repair capacity of mitochondrial genome.

Several NER-associated proteins have been found to be located in mitochondria and involved in the oxidative mtDNA damage repair, including CSA, CSB and Rad23a (26,37), Specifically, Rad23a was recruited to mitochondria upon oxidative DNA damage (37). Thus, XPD may stimulate or participate in mtDNA repair pathway(s) through interaction with these factors. However, lack of direct association observed between XPD and CSA or CSB in our co-IP assays (Supplementary Figure S4), which is consistent with the published literature where the interaction of CSB has only been demonstrated with XPG (43), suggests that XPD may function independently of CSA or CSB proteins in mitochondrial repair of oxidative DNA damage.

We next searched for other factors that may corroborate with XPD in the repair of mitochondrial oxidative DNA damages by immunoprecipitation together with mass spectrometric analysis on mitochondrial fraction, and demonstrated that TUFM interacts with XPD which was further validated by co-IP analysis. TUFM is a mitochondrial elongation factor involving mitochondrial protein translation and biosynthesis (44). It promotes the codon-directed binding of the aa-tRNA to the A-site of the ribosome and protects the tRNA from hydrolysis through forming a ternary complex with GTP and aminoacylated (aa)-tRNA Additionally, it has also been implicated in autophagy, oncogenesis, oxidative phosphorylation and protein quality control (45–48). By formaldehyde cross-linking experiment, TUFM was found to be closely contact with mtDNA, suggesting that TUFM may have some functions on mtDNA such as mtDNA repair in addition to its translation role (49). This is supported by our findings that a markedly increased level of mitochondrial CD and a significantly decreased capacity for repairing mitochondrial oxidative DNA damages were observed after TUFM expression in mitochondria was suppressed. Thus, similar to its interacting partner XPD, TUFM also plays a role in oxidative mtDNA damage repair. But how TUFM participates in mtDNA damage repair is unclear. There are reports showing that TUFM could promote autophagy during viral infection (46,50). It is likely that TUFM knock-down may increase the mtDNA mutation rate indirectly through a reduced autophagy response to oxidative stress. However, the precise functionality of XPD-TUFM interaction and their coordinated activities under oxidative stress condition are worthy of further investigation.

Mitochondria are intracellular organelles responsible for ATP production by enzymes of the electron transport chain located in the inner mitochondrial membrane and, therefore, are the primary source of ROS in the cells (51). Because of its close proximity to mitochondrial membrane as well as limited mtDNA repair capacities, oxidative stress is constitutively elevated in mitochondria. Thus, deficiency of proteins such as XPD that are critical in oxidative mtDNA damage repair will eventually lead to mitochondrial dysfunction and the development of mitochondria-related diseases. This is further supported by the fact that both XPD-deficient human disorders and mitochondrial dysfunction-related diseases possess the common phenotypes such as mental retardation, neurodegeneration, aging and tumor predisposition (7,34). Collectively, our findings provide the strong evidence that XPD is involved in oxidative mtDNA damage repair and plays an essential role in maintaining mitochondrial genome stability.

## SUPPLEMENTARY DATA

Supplementary Data are available at NAR Online.

SUPPLEMENTARY DATA
