# Supplementary material for: XPD localizes in mitochondria and protects the mitochondrial genome from oxidative DNA damage
Source: Nucleic Acids Res. 2015 May 12;43(11):5476–88. doi: 10.1093/nar/gkv472 (PMC4477675; doi:10.1093/nar/gkv472)
Supplement: SUPPLEMENTARY DATA [file supp_43_11_5476__index.html]

XPD localizes in mitochondria and protects the mitochondrial genome from oxidative DNA damage — XPD localizes in mitochondria and protects the mitochondrial genome from oxidative DNA damage — SUPPLEMENTARY DATA 

# XPD localizes in mitochondria and protects the mitochondrial genome from oxidative DNA damage

## SUPPLEMENTARY DATA

- SUPPLEMENTARY DATA
- SUPPLEMENTARY DATA
- SUPPLEMENTARY DATA
- SUPPLEMENTARY DATA
- SUPPLEMENTARY DATA
