# Supplementary material for: XPD localizes in mitochondria and protects the mitochondrial genome from oxidative DNA damage
Source: Nucleic Acids Res. 2015 May 12;43(11):5476–88. doi: 10.1093/nar/gkv472 (PMC4477675; doi:10.1093/nar/gkv472)
Supplement: SUPPLEMENTARY DATA [file supp_gkv472_nar-03465-d-2014-File010.docx]

**SUPPLEMENTARY FIGURE LEGENDS**

Supplementary Figure 1. (A) Long range QPCR results of full length mitochondrial DNA (mt-FL) in shControl and shXPD U2OS cells and in NHDF and XPD patient fibroblasts at recovery time-points of 0, 6, 12, 24 and 48 h post 0.5 mM H_2_O_2_ treatment for 1 h. (B) The PCR band density was quantified by Image J software and normalized to mt-ND1 PCR band.

Supplementary Figure 2. Quantification data by Image J software for long range QPCR results from the cells treated with 1 mM H_2_O_2_. (A) XPD knock-down U2OS cell and XPD-deficient human fibroblasts. (B) XPD-silenced U2OS cells with reconstitution of WT XPD or XPD helicase mutant. (C)TUFM knock-down U2OS cells.

Supplementary Figure 3. Long range QPCR results and quantification data of full length mitochondrial DNA (mt-FL) in siControl and siTUFM U2OS cells post treatment with 0.5 mM H_2_O_2_ for 1 h.

Supplementary Figure 4. Results of interactions of XPD with CSA or CSB examined by co-IP assay. (A)Interaction between XPD and CSA was analyzed by Western blotting using anti-CSA antibody (Santa Cruz, sc-376981). (B)For testing the interaction of XPD with CSB, anti-XPD antibody (Cell Signal) was used to pull-down endogenous CSB in the lysate from HEK293 cells. The immunoprecipitated protein was visualized by Western blotting analysis using antibody against CSB (Santa Cruz, sc25370)
