# Supplementary figures and images for: XPD localizes in mitochondria and protects the mitochondrial genome from oxidative DNA damage
Source: Nucleic Acids Res. 2015 May 12;43(11):5476–88. doi: 10.1093/nar/gkv472 (PMC4477675; doi:10.1093/nar/gkv472)

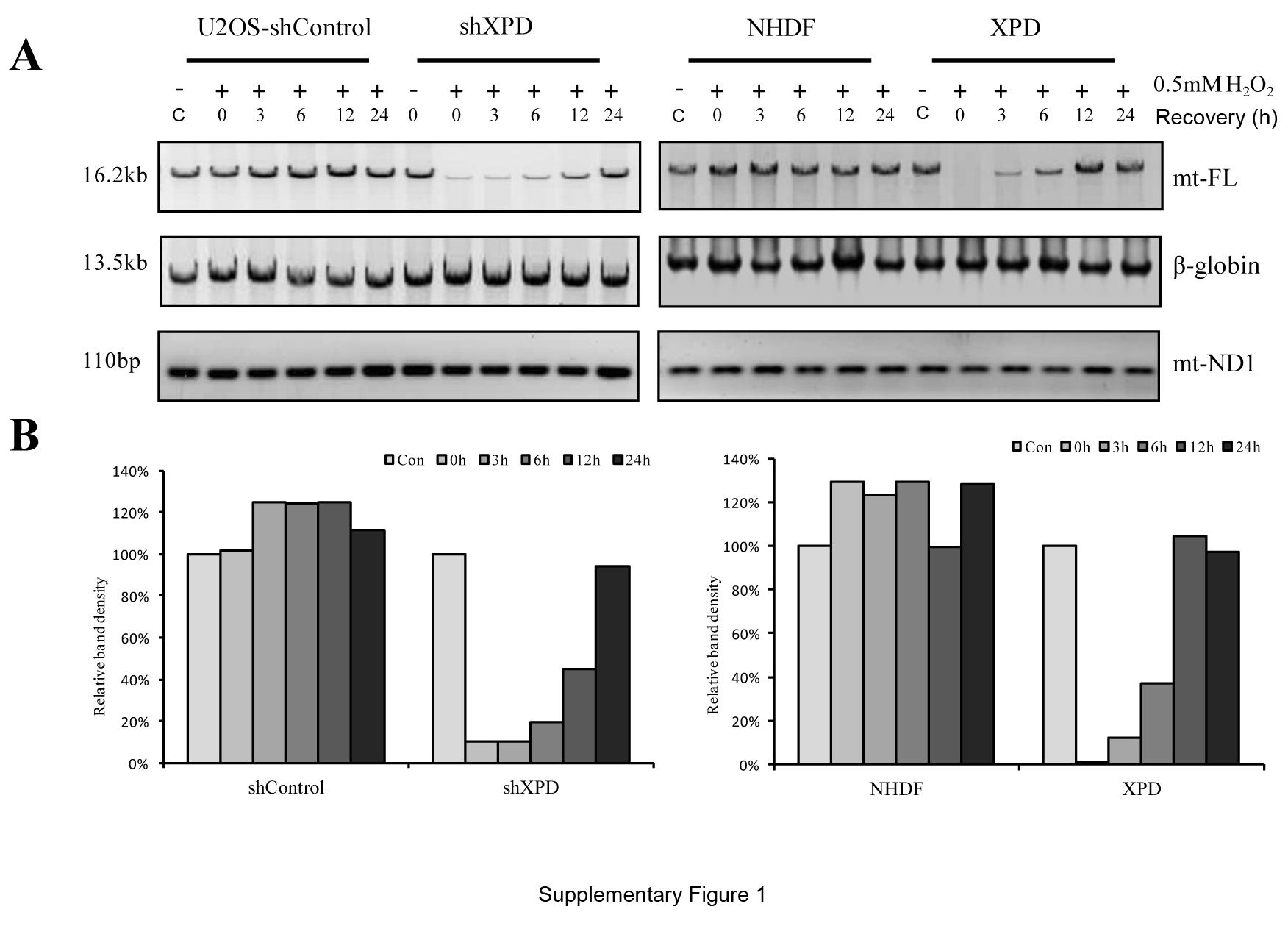

Supplement: SUPPLEMENTARY DATA [file supp_gkv472_nar-03465-d-2014-File011.jpg]

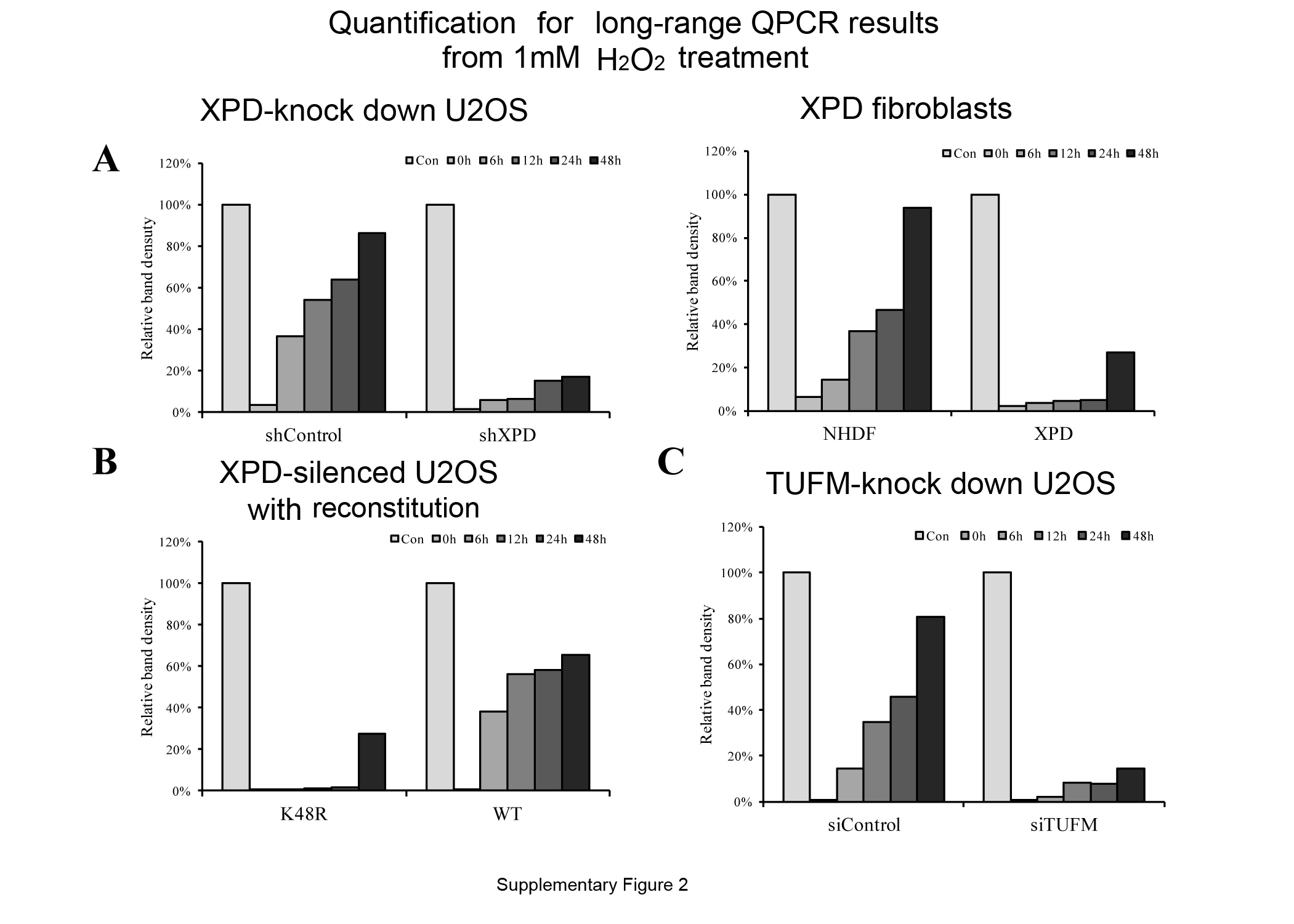

Supplement: SUPPLEMENTARY DATA [file supp_gkv472_nar-03465-d-2014-File012.jpg]

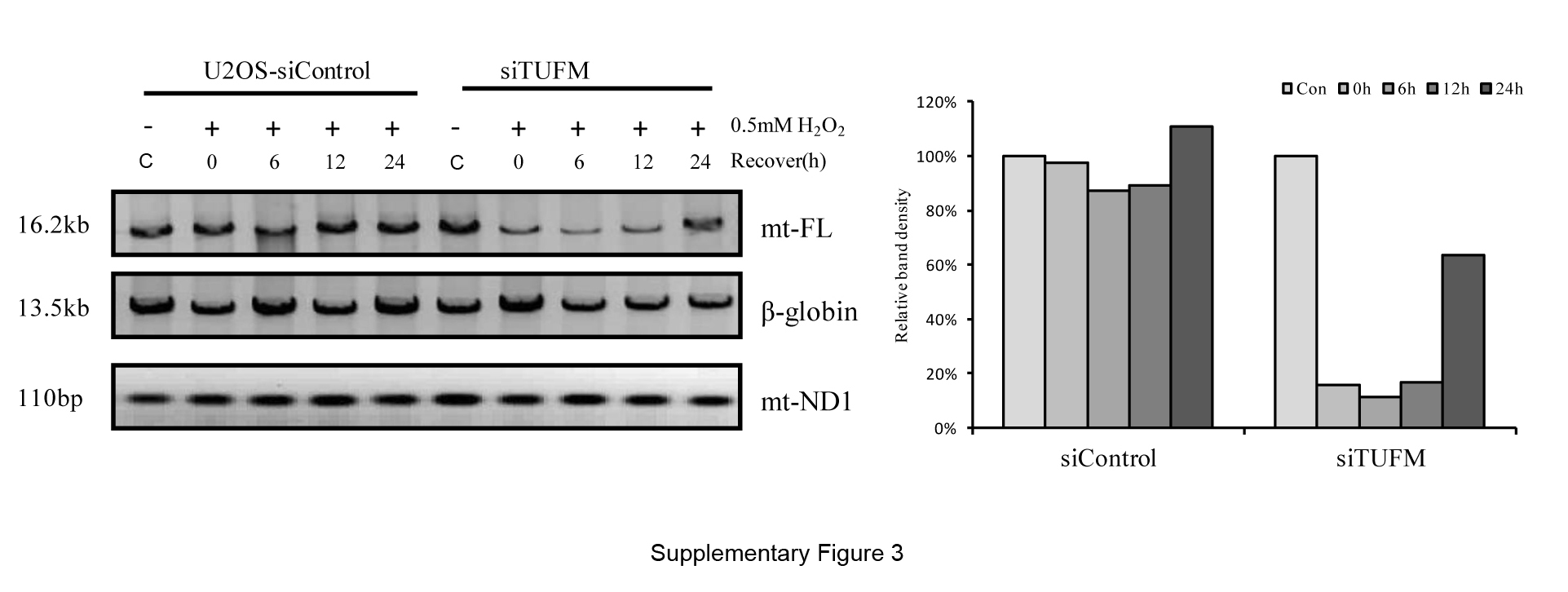

Supplement: SUPPLEMENTARY DATA [file supp_gkv472_nar-03465-d-2014-File013.jpg]

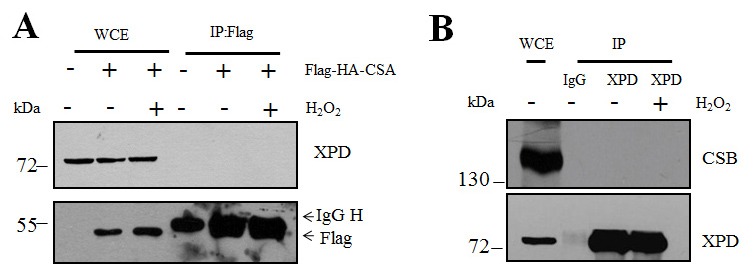

Supplement: SUPPLEMENTARY DATA [file supp_gkv472_nar-03465-d-2014-File014.jpg]
